# The mitochondrial permeability transition phenomenon elucidated by cryo-EM reveals the genuine impact of calcium overload on mitochondrial structure and function

**DOI:** 10.1038/s41598-020-80398-8

**Published:** 2021-01-13

**Authors:** Jasiel O. Strubbe-Rivera, Jason R. Schrad, Evgeny V. Pavlov, James F. Conway, Kristin N. Parent, Jason N. Bazil

**Affiliations:** 1 Pharmacology and Toxicology, Michigan State University, East Lansing, MI 48824 USA; 2 Biochemistry and Molecular Biology, Michigan State University, East Lansing, MI 48824 USA; 3 Basic Science and Craniofacial Biology, New York University, New York, NY 10010 USA; 4 Structural Biology, University of Pittsburgh School of Medicine, Pittsburgh, PA 15261 USA; 5 Physiology, Michigan State University, East Lansing, MI 48824 USA

**Keywords:** Cryoelectron microscopy, Mitochondria, Energy metabolism, Calcium signalling

## Abstract

Mitochondria have a remarkable ability to uptake and store massive amounts of calcium. However, the consequences of massive calcium accumulation remain enigmatic. In the present study, we analyzed a series of time-course experiments to identify the sequence of events that occur in a population of guinea pig cardiac mitochondria exposed to excessive calcium overload that cause mitochondrial permeability transition (MPT). By analyzing coincident structural and functional data, we determined that excessive calcium overload is associated with large calcium phosphate granules and inner membrane fragmentation, which explains the extent of mitochondrial dysfunction. This data also reveals a novel mechanism for cyclosporin A, an inhibitor of MPT, in which it preserves cristae despite the presence of massive calcium phosphate granules in the matrix. Overall, these findings establish a mechanism of calcium-induced mitochondrial dysfunction and the impact of calcium regulation on mitochondrial structure and function.

## Introduction

Mitochondria regulate cell fate through a variety of means^1–5^. Their extensive networks and dynamic architecture facilitate metabolic signaling to ensure proper cellular function and survival. Mitochondria achieve this by integrating intracellular cues and physiological stimuli to regulate ATP production, metabolite oxidation, calcium signaling, phospholipid and steroid hormone biosynthesis, and mitochondrial fission and fusion processes^6–13^. As such, mitochondria must operate under a range of physiological conditions including transient changes in energy demand, oxidative stress, and moderate calcium overload. For example, in highly metabolic organs such as the heart, brain, and kidney, their response to these conditions is crucial for cell survival^14^. However, in pathological conditions, such as during an ischemia/reperfusion event, mitochondria undergo a phenomenon known as the mitochondrial permeability transition (MPT). MPT is a gateway mechanism for cell death and involves the opening of a non-selective pore that allows small molecules and metabolites up to 1.5 kDa in size to freely diffuse across the inner mitochondrial membrane^15,16^. When the pore is open, the membrane potential is dissipated, there is a loss of respiratory control, ATP is hydrolyzed, and osmotic swelling occurs^5^. The swelling causes inner membrane unfolding, outer membrane rupture, and eventually release of apoptogenic molecules, including cytochrome c (cyt. c) that ends in cell death.

While the consequences of MPT are well appreciated, the molecular composition of the pore is currently unknown. The MPT phenomenon was first observed nearly seven decades ago when early studies in the mid-1950s to early 1960s demonstrated massive mitochondrial swelling under certain conditions^17–21^. These conditions involved calcium overload, high inorganic phosphate concentrations, fatty acids, oxidative stress, and adenine nucleotide pool depletion. Interestingly, acidosis, adenine nucleotides, divalent cations (e.g., Mg^2+^, Mn^2+^, Ba^2+^ and Zn^2+^), and some metabolic cofactors^22^ prevent pore opening. In the late 1970s, Haworth and Hunter introduced the term *permeability transition* and highlighted two important points: 1) pore opening is triggered by calcium, and 2) it is closed when calcium is removed from the environment^23^. Their results were later confirmed by Crompton et al.^24^ who further proposed that the pore may have a protein identity with some physiological role^5,16,23,25^. Soon after, other pioneering studies demonstrated such phenomenon was involved in many pathophysiological diseases and conditions such as neurological disorders, aging, response to toxins, cancer, muscular dystrophy, and ischemia–reperfusion injury^14,26–29^. Despite the well-known effects of calcium overload on mitochondrial function, the specific details remain a mystery.

As of now, the current dogma of mitochondrial calcium overload is that mitochondrial dysfunction arises from the opening of a calcium-dependent, free radical sensitized, and proteinaceous molecular pore whose molecular identity thus far remains elusive. Unfortunately, efforts to identify the gene products responsible have been a rollercoaster ride of misleading discoveries and dashed hopes^30–32^. Instead of focusing on the pore, we sought to investigate the consequence of excessive calcium overload on a population of isolated mitochondria by analyzing cryo-electron microscopy (cryo-EM) time-course data. This powerful imaging technique was coupled with high-resolution respirometry and spectrofluorimetry to structurally analyze the effect of calcium overload on mitochondrial function. We identified a novel mechanism that links calcium phosphate granule formation to cristae structural changes, inner membrane fragmentation, and ultimately mitochondrial permeabilization. This mechanism is not mutually exclusive with the current dogma as it integrates many past findings in a concise, overarching theoretical framework. However, our new data add exciting therapeutic targets for mitochondrial-protective therapies.

## Results

### Respiratory inhibition by calcium overload is reversible in low-to-moderate calcium overload

**Figure 1:**
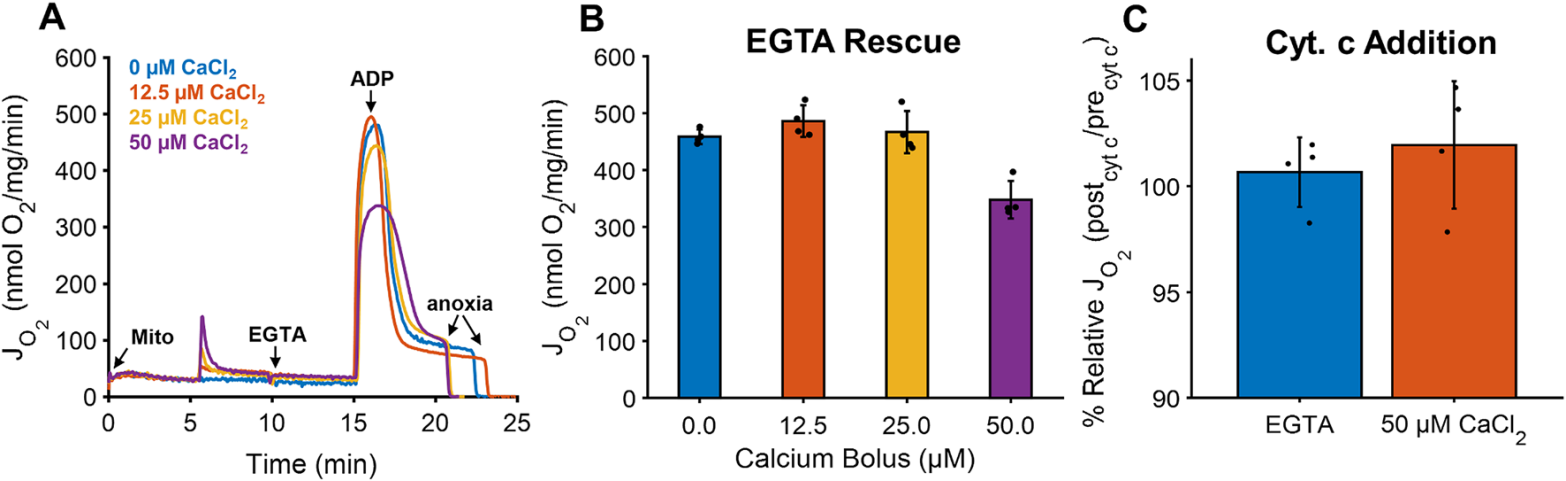
EGTA rescues mitochondrial function at low-to-moderate calcium loads but not high loads. (**A**) Representative traces of ADP-stimulated respiration from calcium loaded mitochondria following the addition of EGTA. Mitochondria (0.1 mg/mL) were energized with 5 mM sodium pyruvate and 1 mM L-malate and exposed to various calcium boluses (0, 12.5, 25, and 50 µM). Five mins after calcium uptake, 1 mM EGTA was added to chelate all calcium in the system. Following an additional 5 min, a bolus of 500 µM ADP was added to induce maximal ADP-stimulated respiration. (**B**) ADP-stimulated mitochondrial respiration is recovered after EGTA addition for all but the 50 µM calcium bolus. (**C**) However, the outer membrane integrity is maintained at the highest calcium dose as shown after the addition of a 10 µM cyt. c. Data are presented as mean ± standard deviation for a sample size of n = 4. Statistical comparisons are made with respect to 0 µM calcium. *Represents a *p* value < 0.01.

While mitochondrial calcium concentrations lower than 100 nmol calcium/mg mitochondria support ATP production^8,10,33^, levels above 500 nmol/mg mitochondria depress oxidative phosphorylation^34–37^. In one of these studies, it was proposed that calcium phosphate precipitates form in the mitochondrial matrix at high calcium loads and reduce ATP production rates by either impeding metabolite transport and diffusion or destabilizing cristae, the functional units of mitochondria. However, the lasting effects of significant calcium accumulation were not explored in either of these studies. To test this, we monitored mitochondrial respiration rates following the addition of the calcium chelator EGTA under various calcium boluses in the range of 0–500 nmol/mg as shown in Fig. 1.

The results in Fig. 1 show that the inhibitory effect of calcium overload is reversible for all but high calcium loads. As expected, the respiratory rates before calcium addition were equal across conditions. After the calcium-dependent transient increase in respiration, respiration remains elevated due to the activation of calcium-sensitive matrix dehydrogenases and sodium/calcium cycling. When 1 mM EGTA was added, the ADP-driven respiratory rates were similar across all conditions except for the highest dose tested.

To rule out that the observed decrease in mitochondrial respiration for the 50 µM CaCl_2_ bolus is due to mPT activation, we assessed outer membrane integrity by exposing mitochondria to an exogenous bolus of 10 µM cytochrome c (Fig. 1C). As shown, mitochondrial respiration did not go above 5% relative to the baseline for either condition confirming that the outer membrane was mostly intact at the highest calcium dose and disputs the notion that the observed detrimental effects on respiration are caused by mPT activation. These results suggest that when calcium overload exceeds a certain threshold, mitochondrial oxidative phosphorylation is irreversibly inhibited. This effect does not involve mitochondrial calpains^34^ and may involve some sort of structural change that lowers ATP production rates. Thus, the effect of calcium overload lies on a spectrum whereby higher levels of calcium result in detrimental changes in mitochondrial bioenergetic pathways.

### CsA preserves the mitochondrial function under high calcium loads

**Figure 2:**
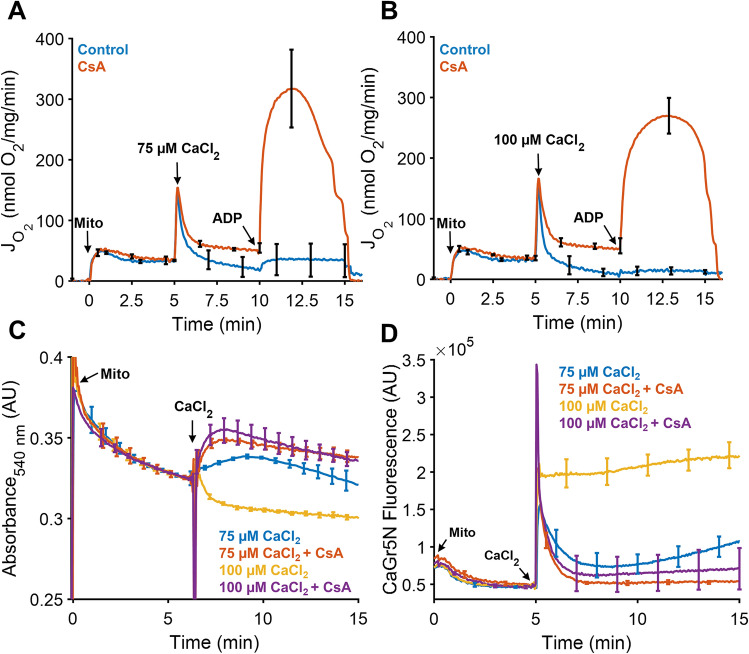
CsA preserves ATP synthesis, increase the absorbance, and enables calcium uptake in a calcium dependent manner. (**A**) and (**B**) The addition of 1 µM CsA prevented a near-total collapse of ADP-stimulated respiration after a bolus of 75 or 100 µM calcium chloride. Mitochondria were energized as described in Fig. 1. (**C**) Mitochondrial swelling was monitored in parallel by quantifying absorbance at 540 nm. Large amplitude swelling was only observed in the 100 µM calcium bolus group when CsA was absent. (**D**) Experimental conditions for calcium uptake were similar except that these experiments were performed in a cuvette open to atmosphere and tracked using the fluorescent probe CaGr5N (1 µM). In the absence of CsA, both calcium boluses were not completely taken up by the mitochondria, while in some instances, mitochondria can uptake calcium followed by release as shown after the addition of a 75 µM calcium chloride bolus. In contrast, CsA enables near-complete calcium uptake of either bolus. Data are presented as mean ± standard deviation for a sample size of n = 3–4.

We then measured mitochondrial respiratory rates during excessive calcium overload by adding a 75 μM or 100 μM calcium bolus in the presence or absence of CsA, a known PTP inhibitor (Fig. 2A,B). In agreement with results from Fig. 1, increasing the extent of calcium overload impairs oxidative metabolism. However, the depressive effects of calcium on ADP-stimulated respiration are much more severe at these higher doses. The respiratory rate after ADP addition drops below 50 nmol O_2_/mg/min after the 75 μM CaCl_2_ bolus and drops below 20 nmol O_2_/mg/min for the 100 μM CaCl_2_ bolus. When CsA was present, this calcium-dependent inhibitory effect is partially mitigated with rates reaching nearly 320 nmol O_2_/mg/min after the 75 μM bolus and 280 nmol O_2_/mg/min for the 100 μM bolus. Therefore, as others have found, CsA partially preserves mitochondrial function in the face of overwhelming calcium overload^31,38–41^. This effect is typically attributed to the ability of CsA to inhibit PTP opening. Our structural data shown in the following sections suggest the existence of a novel protective effect of CsA.

In addition to the respirometry studies, mitochondrial absorbance data obtained in parallel (Fig. 2C) shows that only the 100 µM CaCl_2_ bolus elicited large amplitude swelling, a classic indicator of mitochondrial permeability transition^42^. In contrast, the addition of a 75 µM CaCl_2_ bolus induced an increase in absorbance due to the formation of calcium phosphate granules scattering light at this wavelength^43^. The gradual decrease in absorbance that follows is attributed to mitochondria fragmenting over time in response to the calcium insult. For both CsA-treated groups, the calcium-dependent increase in absorbance was sustained followed by a much slower decrease. However, the decrease in signal is not due to mitochondrial fragmentation but rather due to the inner membrane reorganization and matrix expansion^44,45^ as discussed in later sections of this report. These results are similar to findings from a recent study that looked at the effects of the mitochondria-targeting peptide SS-31 on reducing infarct size of reperfused ischemic hearts^46^.

Our interpretation of the absorbance data is supported by the calcium uptake data shown in Fig. 2D. These data also demonstrate the profound beneficial effects of CsA on mitochondrial calcium sequestration. When CsA was absent, mitochondria were not able to maintain calcium homeostasis and calcium was released into the buffer. For the 75 µM CaCl_2_ challenge, this release was gradual and suggests there is a snowball-like effect in which mitochondria with lower calcium tolerances release their calcium loads and force other mitochondria to take up even more calcium^16,43,47^. This results in additional mitochondria losing their ability to retain calcium, and the process repeats until the entire population is compromised. In contrast, mitochondria were not able to effectively take up and store the 100 µM CaCl_2_ bolus at all when CsA was absent. This level of calcium overload is sufficient to rapidly compromise the entire population in short order.

### Elucidating the effects on calcium overload on mitochondrial ultrastructure

**Figure 3:**
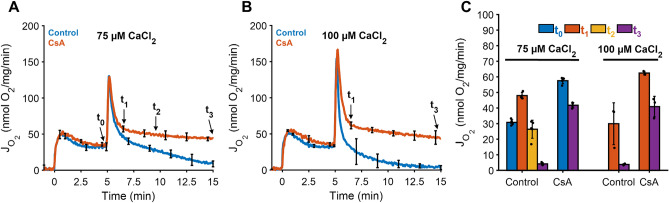
Cryo-EM sample collection protocol and time-points. (**A**) For the 75 µM calcium chloride bolus, 5 µl of the mitochondrial suspension were collected and deposited on the Quantifoil holey-carbon grid at 5 min just before calcium addition (t_0_), approximately 1.5 min after calcium addition (t_1_), 4 min after calcium addition (t_2_), and 10 min after calcium addition (t_3_). (**B**) For the 100 µM calcium chloride bolus, the mitochondrial suspension was sampled at t_1_ and t_3_. In all conditions, mitochondria (0.1 mg/mL) were energized with 5 mM sodium pyruvate with 1 mM L-malate. (**C**) The effect of calcium in the presence or absence of CsA was quantified for each time-point. In the absence of CsA, mitochondrial respiration decreases dramatically as a function of time and the effect is exacerbated at greater calcium loads. In the presence of CsA, mitochondrial respiration was maintained. Data are presented as mean ± standard deviation for n = 3–5 biological replicates.

To capture mitochondria undergoing MPT during calcium overload, we used the sampling scheme shown in Fig. 3. These samples were drawn from a cuvette of isolated mitochondria at the indicated time points and subsequently vitrified in liquid ethane and imaged using cryo-EM. A total of 1345 cryo-EM images were analyzed and organized by sample time-point; before adding calcium (t_0_) and 1.5 min (t_1_), 4 min (t_2_), and 10 min (t_3_) after adding a calcium bolus. We found that many mitochondria shared certain features at each time point and grouped them into 5 stages based on morphology and structure. Each stage represents the transition leading towards complete fragmentation and loss of function in the context of calcium phosphate granules abundance, growth, outer membrane rupture, cristae integrity, and inner membrane fragmentation (Supplementary Table 1). These panels represent the typical process induced by a 75 µM bolus of calcium in a population of isolated mitochondria.

**Figure 4:**
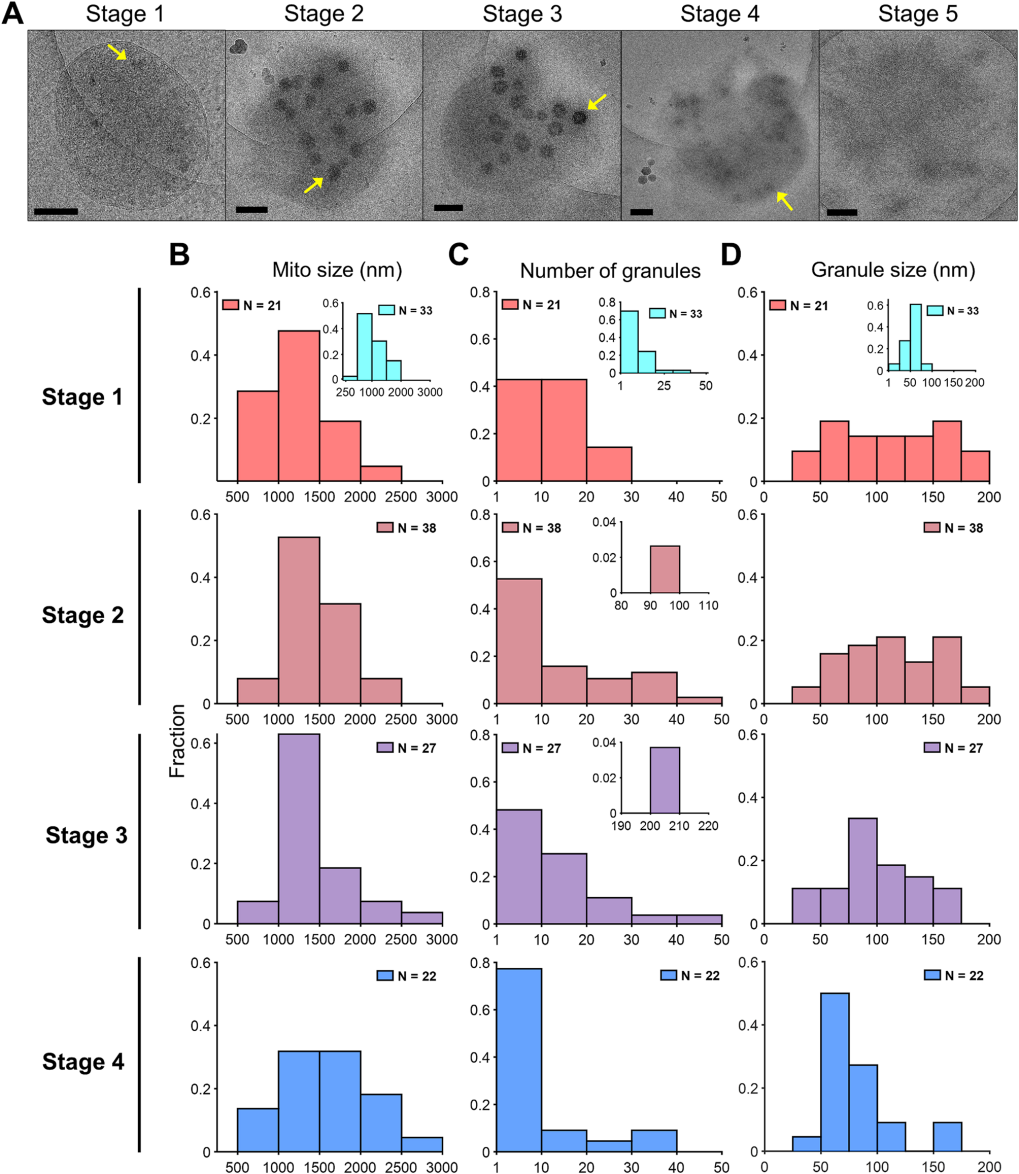
Calcium induces the formation of calcium phosphate granules, outer membrane rupture, and inner membrane evisceration. (**A**) Representative images of mitochondria from stages 1–5 show that calcium induces inner membrane evisceration and outer membrane rupture. The lighter circle is the hole of the Quantifoil carbon grid. Mitochondria typically adhere to the carbon support film that is made hydrophilic after plasma treatment, so they are often either entirely on the carbon or half-on and half-off as shown in these images. The images from stage 2 through 4 contain ice contamination. These ice crystals appear as dark spots with a white fringe or halo outside and below or to the side of the mitochondria. These are easily distinguishable from calcium phosphate granules located in the mitochondrial matrix. Scale bars are 250 nm. The insets for the Stage 1 histograms (**B**–**D**) are histograms calculated from images collected before the addition of a 75 µM calcium bolus. The presence of these granules is due to ~ 4 µM calcium contamination in the respiration buffer. The addition of a 75 µM calcium bolus leads to the formation of much larger and more abundant calcium phosphate granules of various sizes. (**B**–**D**) The mitochondrial sizes and the calcium phosphate granules number and sizes per mitochondrion were further quantified. The mitochondrial size does not change significantly between stages. However, the granule abundance decreases by stage 4. Granules dissolving due to inner membrane fragmentation and loss of membrane potential cause this. The number of images analyzed for each stage is given by n. Note, no histograms for stage 5 are given as this stage consists of completely fragmented mitochondria with no calcium phosphate granules. The data from histograms were obtained from only mitochondria containing calcium phosphate granules. Arrows point to calcium phosphate granules.

Mitochondria in stage 1 have intact inner and outer membranes and are typically round (Fig. 4A and Supplementary Fig. S1). Cristae structures in this set of images are hard to distinguish; however, some are identifiable. Before the addition of a calcium bolus mitochondria are smaller with some calcium granules due to contaminating calcium (~ 4 µM) from buffer solutions as shown by the insets in Fig. 4B–D. The number of calcium phosphate granules is relatively low with sizes averaging less than 100 nm in size. After the addition of 75 µM CaCl_2_, mitochondria begin to fragment and lose bioenergetic competency. The beginning of this process is characterized by stage 2 (Fig. 4 and Supplementary Fig. S2). In this stage, regions of localized outer membrane rupture are observed and are always accompanied by the appearance of calcium phosphate granules. While the size of granules within a mitochondrion does not vary significantly, differences between mitochondria are common and noticeable (Supplementary Fig. S3). During the transition from stage 2 to stage 3, outer membrane definition is lost, and the inner membrane is released. The inner membrane also begins to fragment in this stage. In some instances, calcium phosphate granules are still present indicating that the inner membrane is still energized. However, there are also images of this stage showing granules in the middle of dissolution (Supplementary Fig. S4), suggesting this stage is when depolarization begins. Unexpectedly, the core of the granules appears to dissolve first. In stage 4, the outer membrane is almost entirely gone, and the inner membrane is extensively fragmented. Stage 5 is characterized by the complete fragmentation of the mitochondrial inner membrane and is the dominant stage at the 15 min time point. In this stage, mitochondria are deenergized and contain no calcium phosphate granules.

There were no large differences in mitochondrion sizes between the time points (ranging 500–3000 nm), but there were some clear differences in the size and number of granules (Fig. 4B–D). As mitochondria transition from stage 1 to stage 3, the increase in absorbance shown in Fig. 2C is caused by the increases in numbers and sizes of calcium phosphate granules. In fact, the number of calcium phosphate complexes per mitochondrion reaches a maximum by stage 3 and decreased in the following stage as shown in Fig. 4C. The decreased in size and abundance by stage 4 is due to more complete mitochondrial permeabilization and fragmentation. Hence, for the first time to our knowledge, the MPT phenomenon now has direct visual confirmation of the processes proposed to occur. However, our results elucidate a mechanism that pinpoints cristae remodeling and inner membrane fragmentation as the key determinant of mitochondrial dysfunction as discussed further below.

### CsA preserves the inner membrane, promotes the formation of granules of greater size, and increases the abundance

**Figure 5:**
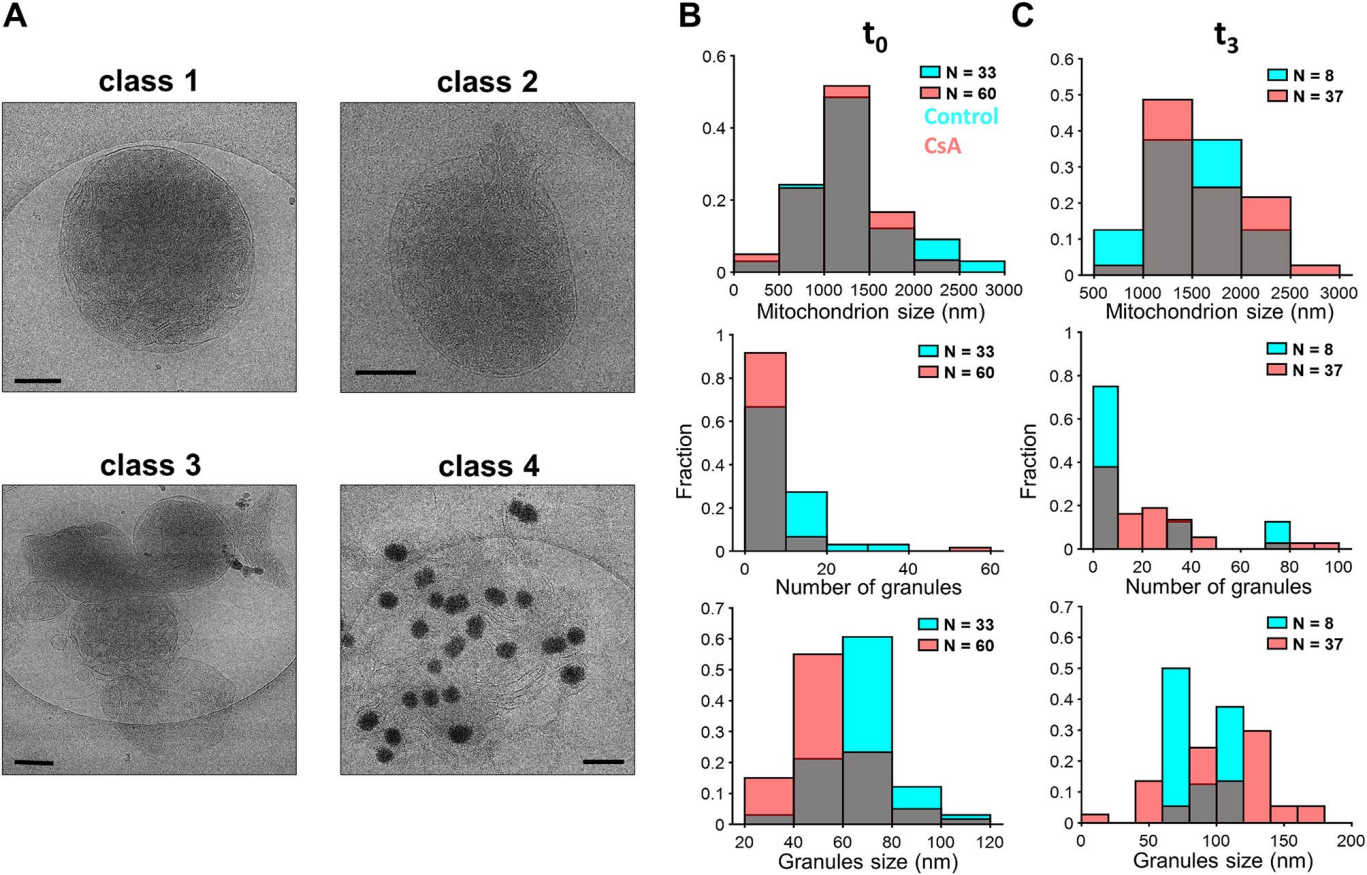
CsA disrupts OMM morphology, causes IMM release, and enhances the number and size of granules. Representative images before the addition of a 75 µM calcium bolus (t_0_) in the presence of 1 µM CsA. Mitochondria were energized with 5 mM sodium pyruvate and 1 mM L-malate. (**A**) CsA induced morphological changes to mitochondria that can be grouped into 4 classes as described in Supplementary Table 2. (**B**) The mitochondrial size, calcium phosphate granules size and number per mitochondrion were quantified for each time-point (t_0_–t_3_) before and after the addition of a 75 µM calcium bolus in the presence or absence of CsA. (**B**) There are no differences in the mitochondrial size of control to CsA-treated mitochondria before the addition of calcium. (**C**) After the calcium addition, the number and size of the granules increased in CsA-treated mitochondria were much larger than in the control mitochondria. Scale bars are 250 nm. n represents the number of images analyzed by the time point for control and CsA treated conditions. Gray zones represent the overlapping points between histograms.

Next, we repeated the calcium overload imaging experiments in the presence of CsA to understand how mitochondrial respiration and calcium handling were preserved from an ultrastructural perspective (Fig. 5 and Supplementary Fig. S5). Like control mitochondria, CsA-treated samples were grouped into 4 classes based on morphology (Supplementary Table 2). However, the classes are not related to a sequence of events like the stages, rather they are descriptive. Many of the images showed normal-looking mitochondria with well-defined inner and outer membranes. These are class 1 mitochondria. Some of these mitochondria contained granules caused by the uptake of low levels of contaminant calcium. In addition, some mitochondria had a condensed inner membrane that was sometimes localized to one side of the mitochondrion. These electron-dense regions are presumably areas of high cristae density. Interestingly, some images showed mitochondria with the outer membrane ruptured with the inner membrane partially or more completely ejected from the mitochondrion. These mitochondria are classified as class 2 mitochondria. In other images, mitochondria were clustered together and are defined as class 3 mitochondria. Lastly, after the calcium treatment, images revealed mitochondria with no outer membrane, large calcium phosphate granules, and the inner membrane spread across the carbon grid. These mitochondria are classified as class 4 mitochondria. Because of morphological changes induced by CsA and calcium addition, the sizes of these mitochondria are larger than mitochondria in the other classes. In addition, mitochondria in this class had granules of heterogeneous sizes between mitochondria but rarely within a single mitochondrion (Supplementary Fig. S6). Despite these radical changes in ultrastructure, the mitochondria remain functionally competent as shown in Fig. 2. The best explanation for this observation is that the cristae junctions and inner membrane integrity are preserved by the CsA treatment.

After calcium addition, the abundance and size of granules per mitochondrion and the mitochondrion size increased in the presence of CsA compared to the control group (Fig. 5). Before the addition of calcium, the average control mitochondrion size was 1320 ± 550 nm, the average granule size of 68 ± 14 nm, and the average number of granules per mitochondrion was 9.7 ± 3.1. Whereas the average CsA-treated mitochondrion size was 1180 ± 400 nm, the average granule size was 55 ± 16 nm, and the average number of granules per mitochondrion was 5.1 ± 2.3. These results show that CsA does not influence any of these parameters before the large calcium bolus was administered. However, after calcium addition, there are noticeable differences between control and CsA-treated mitochondria. The mitochondrial size for control averages 1470 ± 530 nm with a granule size of 90 ± 22 nm and an abundance of 18.0 ± 4.3 per mitochondrion. Whereas CsA-treated mitochondria size average was 1630 ± 400 nm with a granule size of 102 ± 36 nm and an abundance of 26.0 ± 5.1 per mitochondrion. Of note, only a small number of control mitochondria survived by the last time point (Fig. 5C).

### Mitochondrial membrane fragmentation occurs more rapidly at greater calcium loads but is mitigated by CsA

**Figure 6:**
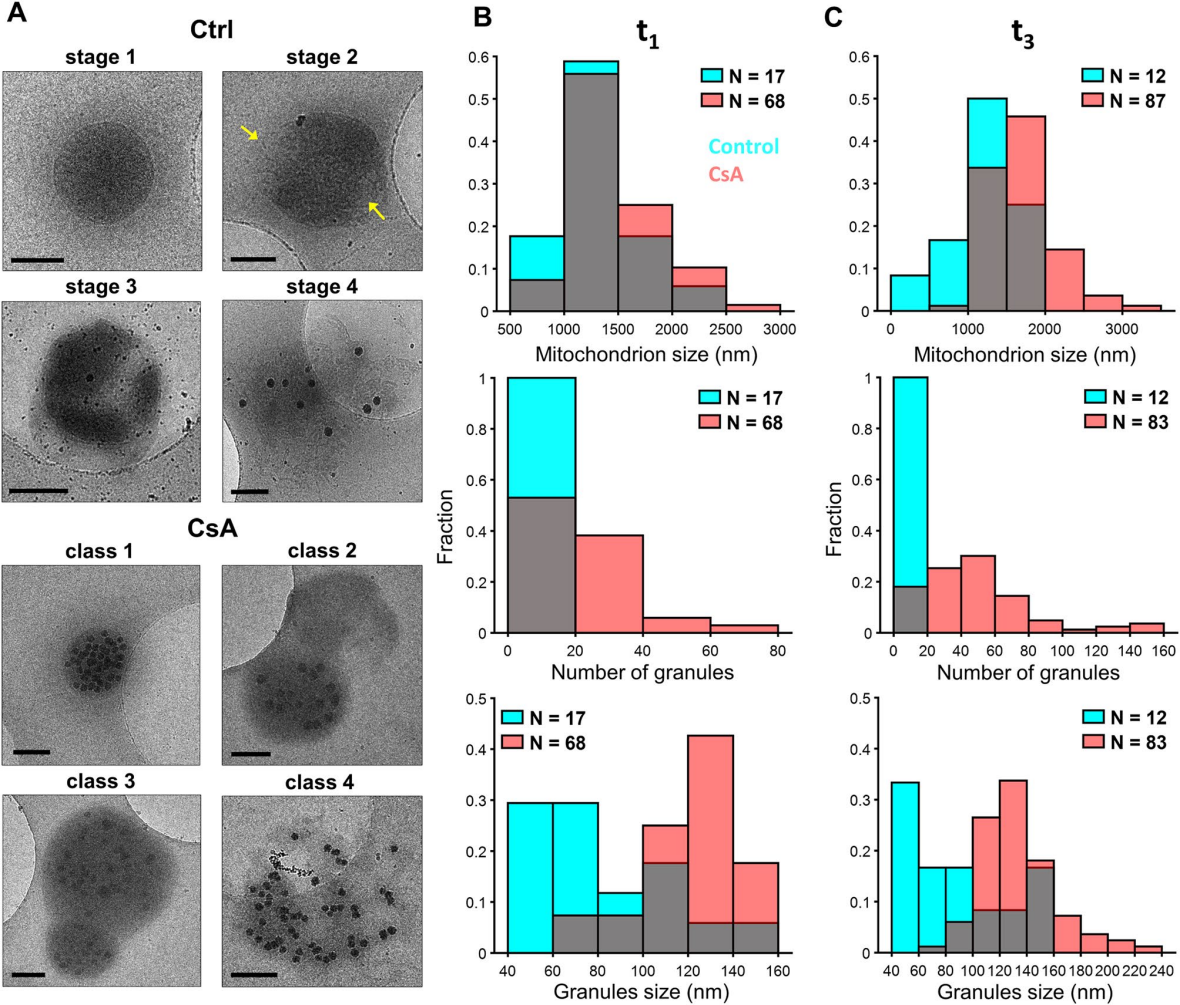
In extreme calcium overload conditions, CsA preserves inner mitochondrial membrane intactness. Representative images before the addition of a 100 µM calcium bolus in the presence or absence of 1 µM CsA. (**A**) The addition of 100 µM calcium led to the formation of granules of various sizes varying between but not within mitochondrion. This large bolus of calcium induces membrane rupture in control mitochondria in multiple location as shown by the arrows. However, mitochondria treated with CsA were protected. (**B**) There are no differences in the average mitochondrial size just after calcium addition between control and CsA-treated mitochondria, but the CsA-treated mitochondria had larger and more abundant granules. (**C**) At 10 min after the addition of calcium, all three measures (mitochondrial size, granule size, granule number) become larger in the CsA-treated mitochondria. In contrast, all three measures decreased in control mitochondria due to membrane fragmentation and evisceration. The number of images analyzed by the time point for control and CsA treated conditions is given by n. Scale bars are 250 nm. Gray zones represent the overlapping points between histograms.

Seeking to understand the observed large amplitude swelling for the control group after the addition of a 100 µM CaCl_2_ bolus, images of control mitochondria were collected after a 100 µM CaCl_2_ addition. Most of the images displayed outer membrane rupture at multiple regions suggesting a rapid expansion of the inner membrane compared to the 75 µM CaCl_2_ bolus (Fig. 6 and Supplementary Fig. S7 and S8). Thus, at this high of a calcium bolus, the morphological changes were caused by what appears to be *bona-fide* permeability transition pore opening. As expected, CsA prevented this rapid expansion and led to the formation of numerous and large calcium phosphate granules. Without CsA, the size and abundance of the granules were noticeably decreased (Fig. 6B). While there were no differences in mitochondrion sizes between treatments shortly after calcium addition (1320 ± 370 nm vs 1490 ± 380), the average control mitochondrial size decreased to 1160 ± 440 nm in the last time point (Fig. 6C). In contrast, the average CsA-treated mitochondrion size increased to 1710 ± 440 nm. The average number of granules in control mitochondria as a function of time was reduced from 9.6 ± 3.1 to 6.6 ± 2.6. The average size of these granules marginally increased from 84 ± 32 to 90 ± 37 nm. Whereas the average number of granules in CsA-treated mitochondria increased from 46.1 ± 6.8 to 107 ± 10 with average sizes increasing from 121 ± 21 to 132 ± 28 nm. These values are greater compared to the values measured after a 75 µM CaCl_2_ bolus was given. This is consistent with CsA increasing calcium accumulation and preserving mitochondrial function even at these high calcium loads. However, the oxygen consumption rate after the 100 µM CaCl_2_ bolus was significantly lowered compared to the 75 µM CaCl_2_ bolus (Fig. 2A,B). Hence, we conclude that calcium induces irreversible effects on mitochondrial function and CsA, although not entirely protective, delays complete loss of function, and allows more calcium uptake.

### Calcium phosphate granules are composed of smaller structural units

**Figure 7:**
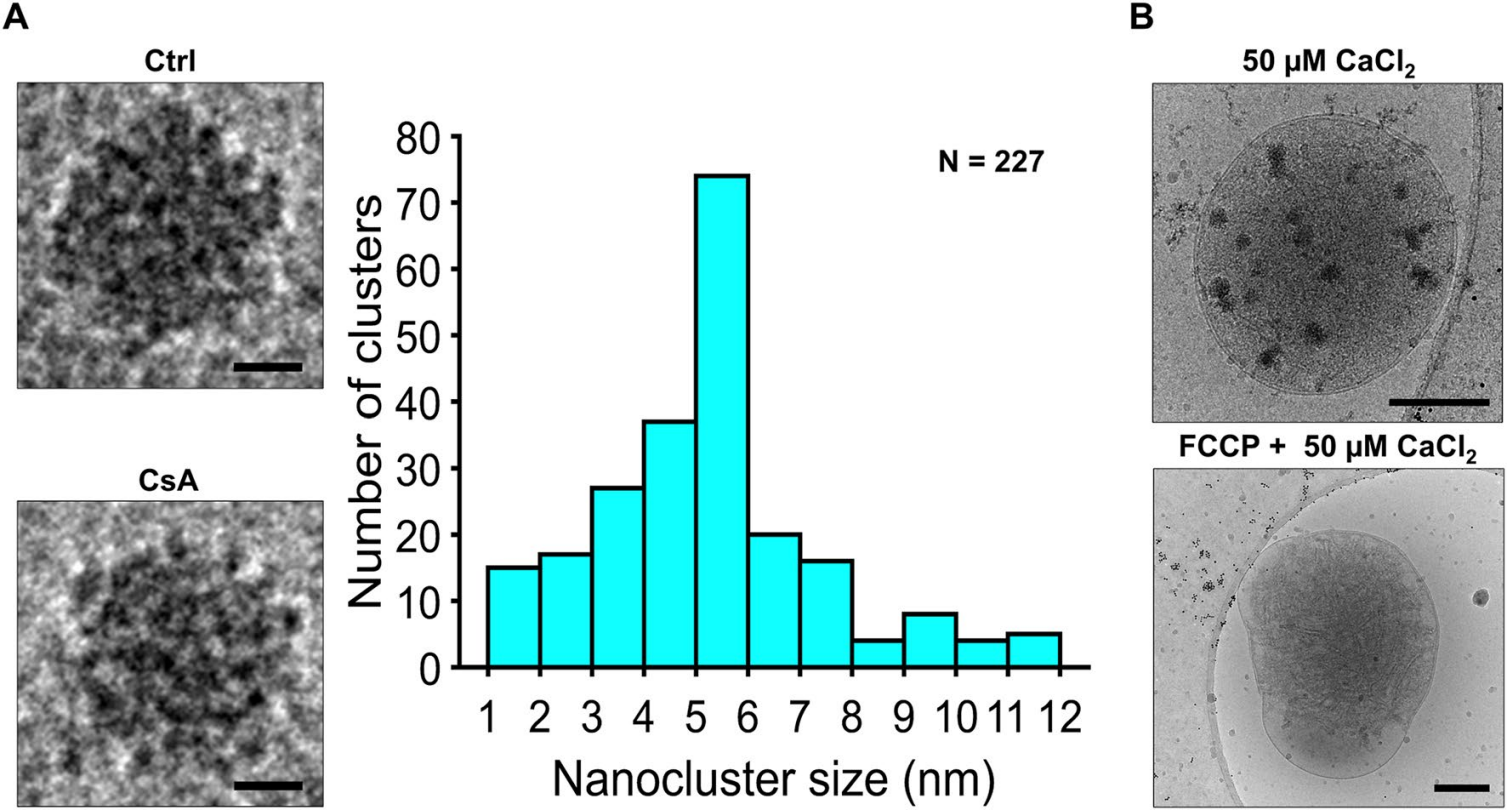
Calcium phosphate granule structure and the effect of membrane potential dissipation. (**A**) To the right, representative images of calcium phosphate granules for each calcium condition in the presence or absence of CsA shows near identical structure. To the left, each granule consists of many individual electron-dense calcium phosphate nanoclusters with a diameter of 5.3 ± 2.1 nm. Scale bars for the granules are 25 nm. (**B**) Representative image of a mitochondrion after the addition of a 50 µM CaCl_2_ bolus contained several visible granules. The uncoupled mitochondrion with 1 µM FCCP before the addition of a 50 µM CaCl_2_ bolus did not contain granules. For the latter, over 100 mitochondria were surveyed, and none contained any granules. The dark spots outside and around the mitochondrion are contaminating ice and gold fiducials approximately 10 nm in diameter. Scale bars are 250 nm.

Calcium phosphate complexes are considered the main component of the mitochondrial calcium sequestration system^34,48,49^. Pioneering studies by Posner and others suggested that amorphous calcium phosphate consists of many smaller spherical elements with a chemical composition of Ca_9_(PO_4_)_6_^50–53^. These elementary units were named as Posner clusters with a diameter ranging from 0.7 to 1.0 nm^52^. In the present study, we lack the image resolution to resolve individual Posner clusters. However, our data show that the calcium phosphate granules are composed of highly electron-dense regions that resemble Posner clusters stacked together forming a higher-order granule structure as shown in Fig. 7A. Our data also reveal that these structures are independent of CsA treatment or sampling time with the only major difference between clusters being their size. We estimated the average calcium load per mitochondrion to be 796 nmol/mg (see Supplementary Results). This value, 796 nmol/mg, is strikingly similar to the expected value of 750 nmol/mg calculated from the calcium uptake data shown in Fig. 2D. Thus, these images yield expected values of calcium uptake and show the overwhelming majority of calcium taken up by mitochondria is stored in these calcium phosphate granules.

### Membrane potential is required to form calcium phosphate granules

Calcium uptake by the mitochondria is highly dependent on the mitochondrial energetic status to regulate ATP production. While the intracellular calcium content is maintained in the 100 nM range, mitochondria gradually accumulates and buffers calcium within the mitochondrial matrix using the membrane potential generated across the inner mitochondrial membrane. This results in calcium phosphate granules formation. To further show this dependency, we used cryo-EM to visualize mitochondria treated with and without the protonophoric uncoupler carbonylcyanide p-trifluoromethoxyphenylhydrazone (FCCP) in the presence of 50 µM CaCl_2_ (Fig. 7B). While the addition of a 50 µM CaCl_2_ generated granules, uncoupled mitochondria did not contained granules in their matrices. The fact that both images in Fig. 7B show intact outer membranes, combined with the results from Fig. 1C, further supports that the absence of granules in uncoupled mitochondria is not due to *bona fide* mitochondrial permeability transition but rather the effect of a collapsed membrane potential and subsequent calcium efflux.

### Absence of granules in mitochondria under extreme high calcium conditions

While most of the isolated mitochondria contained calcium phosphate granules following the calcium addition, a few did not. There are two possible explanations for this phenomenon; either this group of mitochondria is (1) de-energized preventing calcium uptake or (2) they lack mitochondrial calcium uniporters (MCU). Based on the following statistical arguments, the latter is a more likely explanation. Assuming there are 40 MCUs per mitochondrion^8^ with an estimated standard deviation of 20, the probability of randomly selecting a mitochondrion without an MCU channel is 2.3%. This corresponds to 11 mitochondria in our total set of 502 images. In agreement with this estimation, our data show that 17 mitochondria do not possess granules after either calcium bolus was given which corresponds to 3.4% of the number of mitochondria imaged. This percentage is independent of treatment with 3.5% of control mitochondria and 3.6% of CsA treated mitochondria without any calcium phosphate granules. These results also match the respirometry data given in Fig. 2 whereby even after a large bolus of CaCl_2_, some mitochondria are bioenergetically competent and synthesize ATP after the ADP bolus. Assuming that the boluses of calcium were enough to elicit MPT in the mitochondria with an MCU channel, the measured ADP-stimulated respiratory rate increase must be due to mitochondrial lacking an MCU. In line with this observation, the maximum ADP-stimulated respiratory rate for each calcium treatment relative to the maximum rate without calcium (as shown in Fig. 1), is 10.9% ± 5.2 and 4.3% ± 1.1 for the 75 µM and 100 µM CaCl_2_ bolus, respectively. These values are strikingly close to the value estimated from the imaging data.

## Discussion

The current leading hypothesis of calcium-induced mitochondrial dysfunction involves the peptidyl-prolyl *cis–trans* isomerase, cyclophilin D (CypD), interacting with as yet to be identified inner membrane proteins to form the permeability transition pore^24,31,54–56^. When open, the pore results in sustained membrane depolarization, large amplitude swelling, calcium release^23,57^, and loss of mitochondrial respiratory control. CsA can bind to CypD and sequester it so that its interaction with its target is prevented^39^. However, CsA is not fully protective. It is believed that it only increases the calcium threshold required to open the pore. This idea is based on studies that show CsA increases the calcium retention capacity by nearly 3-fold^58^. While an attractive hypothesis, this model has problems that are easier to explain using a different mechanism. As an alternative, we propose a novel mechanism of action whereby CsA enables robust calcium accumulation in the context of promoting calcium uptake and calcium phosphate granule formation. This mechanism involves the interaction between putative CsA-regulated proteins, and cristae structural proteins to preserve the inner membrane intactness. While the calcium phosphate granules may induce changes in morphology by mechanically disrupting membranes, it is plausible that free calcium interacts with proteins regulating inner membrane and cristae maintenance (namely the optic atrophic factor 1 and the mitochondrial contact site and cristae organizing system; known as OPA1 and MICOS) or additional regulators of this system. For instance, the stress-sensing overlapping activity with *m*-AAA protease 1 (OMA1) is a zinc metallopeptidase found in the inner mitochondrial membrane regulating mitochondrial dynamics through OPA1 processing^59–61^. OMA1 is activated under stress conditions including membrane potential dissipation, decreased ATP levels, and oxidative stress, among other insults^61^. Upon activation, OMA1 mediates the proteolytic processing of OPA1 inducing cristae remodeling and cyt. c release^59,62–65^. This mechanism can explain the morphological and functional changes included by calcium overload that we observed in our cryo-EM images and bioenergetics data.

Indeed, we demonstrated that calcium overload impairs mitochondrial ATP production at greater calcium loads and depleting mitochondria of calcium did not fully restore function—indicating an irreversible component. These data revealed an underappreciated energetic consequence of calcium overload on the mitochondrial function that supports a direct role of the mitochondrial calcium buffering system. In cardiac tissue, the steady-state cycling of calcium across plasma membranes maintains cytosolic calcium levels at ~ 100 nM during diastole; however, the peak calcium concentration in the cytosol during systole can rise to the low micromolar range^9,33,66^. Whether the mitochondria can respond to these transient changes to meet metabolic demand is a subject of debate (reviewed in^9^) that revolves around the mitochondrial calcium uniporter (MCU) being unable to approach maximum flux rates in the transient rise of cytosolic calcium due to its low affinity for calcium^67^. Alternative hypotheses regarding calcium microdomains have been proposed in an attempt to argue in favor of significant mitochondrial calcium uptake during systole^68–72^; however, direct imaging studies do not support this^66,73^. A recent study by Wescott et al. found that physiological cytosolic calcium transients cause a gradual, step-wise increase in matrix calcium concentration per beat rather than large transient peaks^8^. They also showed that at high pacing rates, the matrix calcium concentration did not change any further. Further studies are required to determine whether these results are due to equal influx and efflux of calcium per cycle or due to calcium buffering. At this point, it is possible that calcium buffering in the form of calcium phosphate granule formation becomes relevant. In a separate study, calcium phosphate granules were observed in the matrix near cristae junctions in a variety of different eukaryotic cells under physiological conditions^48^. Given the relevance of calcium in bioenergetics, the presence of these calcium deposits may exert some degree of control over mitochondrial signaling and metabolism.

**Figure 8:**
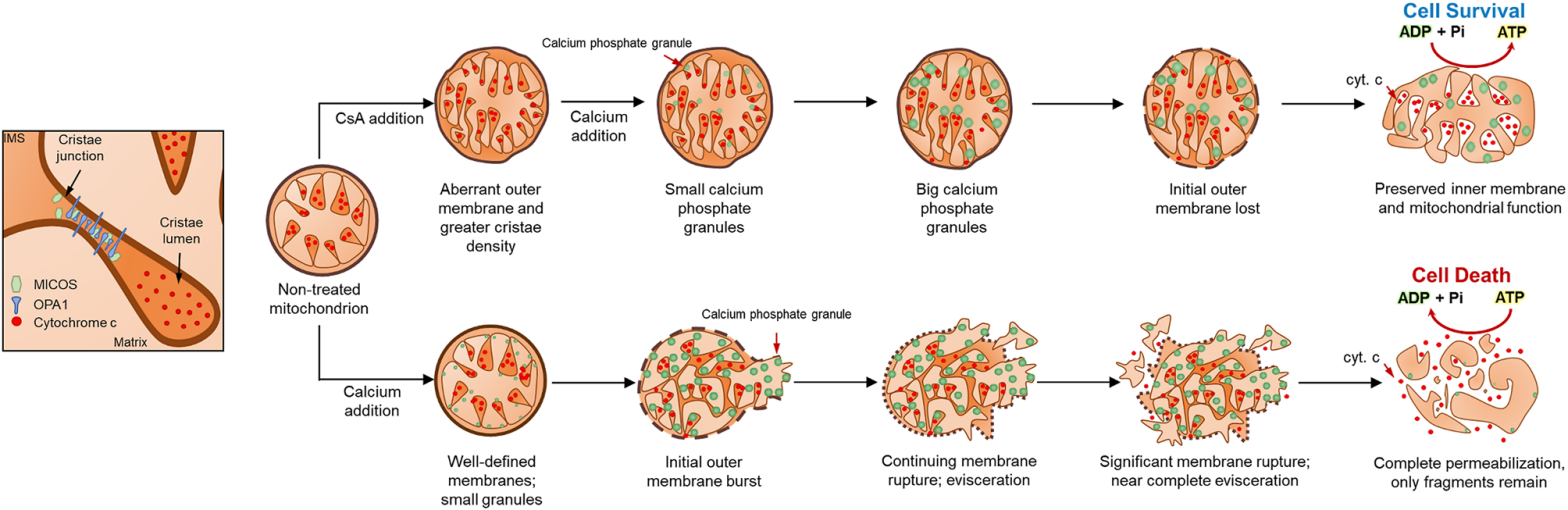
Schematic representation of calcium overload leading to mitochondrial fragmentation and permeabilization. In energized mitochondria, the mitochondrial membrane potential creates the driving force for calcium to accumulate in the mitochondrial matrix. The accumulation and growth of these complexes induces mitochondrial swelling that leads to outer membrane rupture, inner membrane fragmentation and cyt. c release. This causes membrane potential dissipation and induces the calcium-phosphate complex disassembly. CsA, however, alters the membrane morphologies and allows for robust calcium uptake after the addition of a calcium bolus. The inner mitochondrial membrane remains tethered and the cristae junctions intact. This avoids cyt. c remodeling and preserves the bioenergetic status despite the calcium effects on mitochondrial respiration. The drawing was created with Microsoft PowerPoint for Microsoft 365 MSO (16.0.13231.20250) 64-bit.

In Fig. 8, we present a model that accounts for various characteristics of membrane fragmentation before the MPT onset. This model integrates findings from our cryo-EM analysis with mitochondrial function and recapitulates the effects of calcium on the mitochondrial structure. Based on our findings, we believe that changes in mitochondrial ultrastructure can explain the loss of function in calcium overload as well as the protective effects of CsA. Our results suggest that mitochondrial outer membrane rupture and inner membrane fragmentation are *caused* by calcium overload whereas the formation of granules is a *consequence* of calcium uptake and accumulation. In the present study, the detrimental effects of calcium overload on mitochondrial function are mitigated when CsA is present. Regardless of the calcium bolus, the number and size of granules in CsA-treated mitochondria increased, suggesting that CsA increases the mitochondrial calcium buffering capacity, thus explaining why CsA allows robust calcium uptake and increases the threshold for permeability transition pore activation^31,39,41,74^.

To interpret these results, we sought confirmation of our findings from work by others. A study by Pinton’s group^40^ studied the effect of calcium overload on mitochondria in HeLa cells. Exposing HeLa cells to the ionophore ionomycin resulted in mitochondrial network fragmentation. However, in the presence of CsA, the mitochondrial network condensed and maintained its integrity after ionomycin treatment. Another study looking at mitochondrial swelling using light transmittance in a single mitochondrion showed that calcium induces mitochondrial swelling in a concentration-dependent fashion^75^. CsA decreased this effect in a calcium-dependent manner, which led the authors to conclude that either CsA induces mitochondria shrinkage or calcium accumulation induces light scattering. We show that CsA increases the absorbance in a calcium-dependent manner and induces changes in mitochondria ultrastructure including condensed inner membranes and loss of outer membrane. Therefore, our results are consistent with these studies but quantitatively describe the ultrastructural changes associated with calcium overload and how these changes are linked to mitochondrial function.

A major challenge in this study is the lack of cristae structural definition in our set of images. As dynamic structures, cristae are the functional units of mitochondria that lock cyt. c in the cristae lumen and provide sufficient membrane surface area to sustain oxidative phosphorylation at high rates^64,76^. Under certain conditions when the cristae junctional width is enlarged, cyt. c escapes the lumen and causes loss of mitochondria function and cell death^77,78^. While the expected outcomes during calcium overload were addressed, intricate details of the cristae structure including junction width, length, density, and shape must be incorporated to better understand the implications of cristae remodeling as key mediators in mitochondrial function. While energy-dispersive X-ray spectroscopy (EDX) was not included in our studies, others have determined these granules are majorly composed of calcium and phosphorous^48,79^. Earlier studies looking at calcium phosphate granule composition relied on staining, fixing, or dehydrating samples, introducing artifacts which makes them less reliable^80–84^. More recently, changes in mitochondrial structure were analyzed using high-pressure techniques and freeze-substitution to minimize sample structural distortion resulting from fixation or dehydration^79^. However, details such as granule space distribution and structure are not as well defined with this method relative to the latest advanced cryo-EM techniques. Hence, visualization of the mitochondria in 3D by cryo-electron tomography (cryo-ET) would be an avenue for future studies to address. Nonetheless, our finding that CsA preserves the inner membrane integrity suggests that cristae remodeling and cyt. c release from the cristae lumen is likely avoided. This poses a new approach by which therapies targeting cristae remodeling can be identified to prevent pathological mitochondrial dysfunction leading to tissue injury.

## Materials and methods

### Ethical approval

This work conformed to the National Institutes of Health’s Guide for the Care and Use of Laboratory Animals and was approved by Michigan State University’s Institutional Animal Care and Use Committee.

### Mitochondria isolation and protein quantification

Cardiac mitochondria were isolated from guinea pig hearts using differential centrifugation as described in Wollenman et al.^85^ Briefly, Hartley albino guinea pigs weighing 350–450 g (4–6 weeks) were injected with heparin (500 units/mL) into the intraperitoneal cavity to prevent blood clotting during the cardiac mitochondrial isolation. Before heart removal, the animals were deeply anesthetized with 4–5% isoflurane. Prior to decapitation by guillotine, a noxious stimulus (paw pinch and eyelid reflex) confirmed the animals were fully sedated. After decapitation, a thoracotomy was performed. The heart was then perfused with cold cardioplegia solution and homogenized as described previously^85^. Mitochondrial protein content was quantified using the BIO-RAD Bovine Serum Albumin (BSA) Standard Set Kit and the BCA assay. The mitochondrial suspension was diluted to a working concentration of 40 mg/mL and kept on ice for the duration of the experiment (4–8 h). Substrate stock solutions were neutralized to pH 7.0.

### Mitochondrial quality control

The mitochondrial quality was determined using an Oxygraph 2 k (Oroboros Instruments Corp., Innsbruck, Austria) under constant stirring. The O2k chambers were loaded with 2 mL respiratory buffer containing 130 mM KCl, 5 mM K_2_HPO_4_, 20 mM MOPS, and 1 mM MgCl_2_, 1 mM EGTA, 0.1% (w/v) BSA at a pH of 7.1 and 37 °C. All subsequent experiments were done using this buffer and temperature. At 0 min, 5 mM sodium pyruvate and 1 mM L-malate were added followed by 0.1 mg/mL mitochondria. Here we defined leak state as the rate of oxygen consumption by mitochondria only in the presence of substrates. At 5 min a bolus of ADP (500 µM) was added to induce maximal ADP-stimulated respiration. Quality was assessed by computing the respiratory control ratio (maximal ADP-stimulated rate divided by the leak rate). Only mitochondria with an RCR value greater than or equal to 16 were used in the experiments.

### Calcium contamination and buffer calcium measurements

The amount of contaminating calcium present in the respiratory buffer was 4.0 µM ± 0.43 µM which comes from reagent impurities^85^. This was measured using a perfectION calcium selective electrode (Mettler Toledo, Columbus, OH). Results were further confirmed using 1 µM calcium fluorescent indicator calcium green 5N (503 nm excitation and 531 nm emission) using an Olis DM245 spectrofluorimeter (Olis, Inc., Bogart, GA, USA).

### Calcium effects on respiration and oxidative phosphorylation

Calcium effects on mitochondrial leak and ADP-stimulated respiration were determined by quantifying changes in leak and ADP-stimulated respiration rates after a calcium challenge in the presence or absence of cyclosporin A (CsA). At 0 min, 5 mM sodium pyruvate, 1 mM L-malate, ± 1 µM CsA, and 0.1 mg/mL mitochondria were injected into each 2 mL chamber containing respiratory buffer. At 5 min, a calcium bolus of either 75 or 100 µM CaCl_2_ was injected. At 10 min, 500 µM ADP was added induce maximal ADP-stimulated respiration. The respiratory buffer contained no EGTA for these studies.

### Calcium effects on the outer membrane integrity

The effect of calcium on the outer membrane integrity was determined by quantifying changes in ADP-stimulated respiration rates after a calcium challenge by the addition of cytochrome c. At 0 min, 5 mM sodium pyruvate, 1 mM L-malate, and 0.1 mg/mL mitochondria were injected into each 2 mL chamber containing respiratory buffer. At 5 min, a calcium bolus of 50 µM CaCl_2_ or ddH_2_O was injected. At 5 min, 1 mM EGTA was added onto each chamber. At 10 min, 500 µM ADP was added to induce maximal ADP-stimulated respiration. After the respiration rate stabilized (mitochondrial ATP production rate, i.e. oxphos, equal to the exogenous ATPase rate), 10 µM cytochrome c was added. To determine outer membrane integrity, the relative oxygen consumption after cytochrome c addition was divided by the relative oxygen consumption before the cytochrome c addition and multiplied by 100 to express them as a percentage.

### Mitochondrial swelling assay

Mitochondrial swelling was quantified by measuring absorbance at 540 nm using an Olis DM245 spectrofluorimeter with a dual-beam absorbance module. At 0 min, 5 mM pyruvate and 1 mM L-malate was added to a polystyrene cuvette with respiration buffer containing ± 1 µM CsA followed by the addition of 0.1 mg/mL mitochondria. At 5 min, a 75 or 100 µM CaCl_2_ bolus was added and the absorbance was recorded for a total of 15 min. The minimum absorbance signal was determined by adding the uncoupler FCCP (1 µM) and the channel forming peptide Alamethicin (10 µg/mg). To normalize the raw traces, we used the minimum absorbance value followed by the absorbance just before the addition of a calcium bolus.

### Calcium uptake dynamics

Calcium uptake dynamics were quantified using the fluorescent dye, calcium green 5N (CaGr5N). Fluorescence was measured using an Olis DM245 spectrofluorimeter in a closed chamber without interruption of the recording. The sampling rate was 1 Hz with an integration time of 0.25 s, and an automated shutter system was used to minimize phototoxicity. The dye was excited at 506 nm and the emission recorded at 531 nm. To minimize variability in dye concentration, 1 µM CaGr5N was added to 50 mL stocks of respiration buffer as opposed to adding small volumes to the 2 mL assay volume. At 0 min, ± 1 µM CsA, 5 mM sodium pyruvate and 1 mM L-malate, and 0.1 mg/mL mitochondria were added to a polystyrene cuvette constantly stirred at 600 rpm. At 5 min, a bolus of either 75 or 100 µM CaCl_2_ was added by opening the chamber and the fluorescence was recorded for 15 min.

### Cryo-EM sample vitrification and imaging

Isolated mitochondria were suspended at a concentration of 0.1 mg/mL in 2 mL respiration buffer with 5 mM sodium pyruvate and 1 mM L-malate. At the collection times indicated, 5 µL samples were pipetted from the mitochondrial suspension and deposited on Quantifoil R2/2 Holey Carbon grids that had been plasma-cleaned for 20 s using a Fischione Instruments model 1020 plasma cleaner. Grids were blotted to thin the water layer, and subsequently plunged into liquid ethane at room temperature using a manual plunge-freezing device (Michigan State University Physics Machine Shop). Grids were then transferred and stored in liquid nitrogen until imaging. Data for the 75 µM CaCl_2_ experiments were collected in the cryo-EM facility at the University of Pittsburgh School of Medicine using an FEI Polara G2 cryo-electron microscope with a field emission gun operating at 300 kV at nominal magnification of 9400× with a post-column magnification of 1.4× to obtain a ~ 12–10 Å/pixel resolution. Images were recorded on a FEI Falcon 3 direct electron-detecting camera at an electron dose of 0.2 e^−^/Å^2^. Data for the 100 µM CaCl_2_ experiments were collected in the cryo-EM facility at the University of Pittsburgh School of Medicine using a FEI TF20 cryo-electron microscope with a field emission gun operating at 200 kV. The images were collected using a nominal magnification in the range of 5000× on a TVIPS XF416 CMOS camera with a post-column magnification of 1.4× to obtain a 22 Å/pixel resolution. Data for the ± 1 µM FCCP condition at 50 µM CaCl_2_ were collected in the cryo-EM facility on Michigan State University using a Talos Arctica outfitted with a Falcon 3 direct electron-detecting camera. The field emission gun was operating at 200 kV using a magnification of 22,000× at an electron dose of 2.03 e^−^/Å^2^ to obtain a 4.69 Å /pixel resolution. Grids were blotted for 5 s before vitrification in liquid ethane using a Vitrobot Mark IV System from Thermo Fisher Scientific. At these magnifications, the electron dose (e^−^/Å^2^) is low enough to avoid significant sample destruction.

### Calcium phosphate granules, posner’s clusters, and mitochondrial structure quantification

The program EMAN2^86^ was used to quantify the total number of granules for each mitochondrion under each condition from TEM images. A total of 1345 individual mitochondrial images were acquired in the presence and absence of CsA for two calcium treatments. For the 75 µM CaCl_2_ treatment, there were 235 images of control mitochondria and 645 images of CsA-treated mitochondria. For the 100 µM CaCl_2_ treatment, there were 231 images of control mitochondria and 234 images for CsA-treated mitochondria. Mitochondrial and phosphate granule diameters were computed from three averages of two diagonal and one horizontal diameter measurement. Pixel resolution was converted to nanometers based on the magnification level. The fractional area that the calcium phosphate granules occupy per mitochondrion was calculated by multiplying the number of granules within a mitochondrion times the sum of all the granule areas divided by the area of the mitochondrion (N_granules_*A_granules_ / A_mito_). The calcium phosphate nanoclusters (n = 227) were determined by measuring the electron-dense regions located within the granules using ImageJ (NIH, Bethesda, MD, USA).

### Statistics

The Shapiro-Wilks test was used to confirm data normality. All data were analyzed and plotted using either MATLAB 2019a or MATLAB 2019b (Mathworks, Inc., Natick, MA, USA). The data in Figs. 1, 2 and 3 (n = 3–4) and stats presented for the calcium phosphate nanoclusters are presented as mean ± standard deviation. Mitochondrial images with calcium phosphate granules were only included for the histogram analysis (n value in the figures). An unpaired Student’s t test was used to compare the CsA treatment with the control group. An n-way ANOVA was run to determine significant effects between treatments at various calcium loads and different time-points. A *p* value < 0.05 was assumed to be statistically significant.

### Reagents

All reagents were purchased from Sigma-Aldrich unless otherwise stated. Calcium Green-5 N hexapotassium salt was purchased from Thermo Fisher Scientific.

## Supplementary information

Supplementary Information 1.
